# Investigation of the non‐small cell lung cancer patients with bronchus involvements: A population‐based study

**DOI:** 10.1111/crj.13683

**Published:** 2023-08-07

**Authors:** Gang Wang, Yong‐Qiang Ye, Bao‐Long Xie, Xiang‐Min Lai, Sheng‐Peng Zhong

**Affiliations:** ^1^ Department of Thoracic Surgery Ganzhou Tumor Hospital Ganzhou People's Republic of China

**Keywords:** bronchus involvements, non‐small cell lung cancer, prognosis, survival, T category

## Abstract

**Background:**

We aimed to explore the prognostic differences among T1‐4N0‐2M0 non‐small cell lung cancer (NSCLC) patients with bronchus involvements and to validate the T category of these patients in an external cohort.

**Methods:**

Univariable and multivariable Cox analysis was performed to determine the prognostic factors. Kaplan–Meier method with a log‐rank test was used to compare overall survival differences between groups. Propensity score matching method was used to minimize the bias caused by the imbalanced covariates between groups.

**Results:**

A total of 169 390 eligible T1‐4N0‐2M0 NSCLC cases were included. There were 2354, 3367, 1638, 75, 87 585, 42 056, 19 246, and 13 069 cases in the group of superficial tumors of any size with invasive component limited to bronchial wall (T1‐bronchus), tumors involving main stem bronchus ≥2 cm from carina (T2‐main bronchus [≥2 cm]), tumors involving main stem bronchus <2 cm from carina (T2‐main bronchus [<2 cm]), tumors with carina invasion (T4‐carina), T1, T2, T3, and T4, respectively. Multivariable Cox analysis indicated that T1‐bronchus patients had the best prognosis; T2‐main bronchus (≥2 cm) and T2‐main bronchus (<2 cm) patients had similar prognosis both in the entire cohort and in several subgroups. Survival curves showed that T1‐bronchus and T1 patients had similar survival rates; the survivals of T2‐main bronchus patients regardless of the distance from carina were comparable to those of T2 patients, and the survivals of T4‐carina patients were also similar to those of T4 patients.

**Conclusions:**

Our results validated and supported the current T category for the patients with bronchus involvements, which might provide certain reference value for the revisions of T category in the next version of the tumor‐node‐metastasis stage classification.

## Graphical abstract

Core findings: Our results validated and supported the current T category for the NSCLC patients with bronchus involvements. This study might provide certain reference value for the revisions of T category in the next version of the NSCLC TNM stage classification.

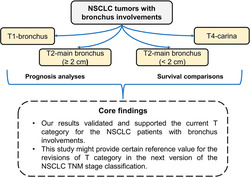

## 1 INTRODUCTION

Lung cancer is the leading cause of cancer‐related mortality worldwide,^1^, ^2^ which consists of non‐small cell lung cancer (NSCLC) and small cell lung cancer.^3^, ^4^ The tumor‐node‐metastasis (TNM) stage classification, an important indicator proposed to assess disease severity and estimate individual survival, has been updated every few years.^5^, ^6^, ^7^, ^8^

Bronchus involvements is a non‐sized T descriptor in the latest NSCLC TNM stage classification.^5^, ^6^ Superficial tumors of any size with invasive component limited to bronchial wall and with or without proximal extension to the main stem bronchus are assigned to T1 category (T1‐bronchus).^5^, ^6^ Tumors involving main stem bronchus ≥2 cm from carina are assigned to T2 category (T2‐main bronchus [≥2 cm]), and those involving main stem bronchus <2 cm from carina are also classified as T2 category (T2‐main bronchus [<2 cm]),^5^, ^6^ which is a T3 descriptor in the 7th edition of the TNM stage classification.^7^, ^8^ In addition, tumors with carina invasion are defined as T4 category (T4‐carina).^5^, ^6^

The current TNM stage classification must be applicable to individual databases, and no external validation study has been performed on the T category of lung tumors with bronchus invasion. Herein, the current study analyzed the data of NSCLC with bronchus involvements deposited in the Surveillance, Epidemiology, and End Results (SEER) program, in an effort to figure out the prognostic disparities and further validate the current T category of these patients.

## 2 MATERIALS AND METHODS

### 2.1 Included patients

**FIGURE 1:**
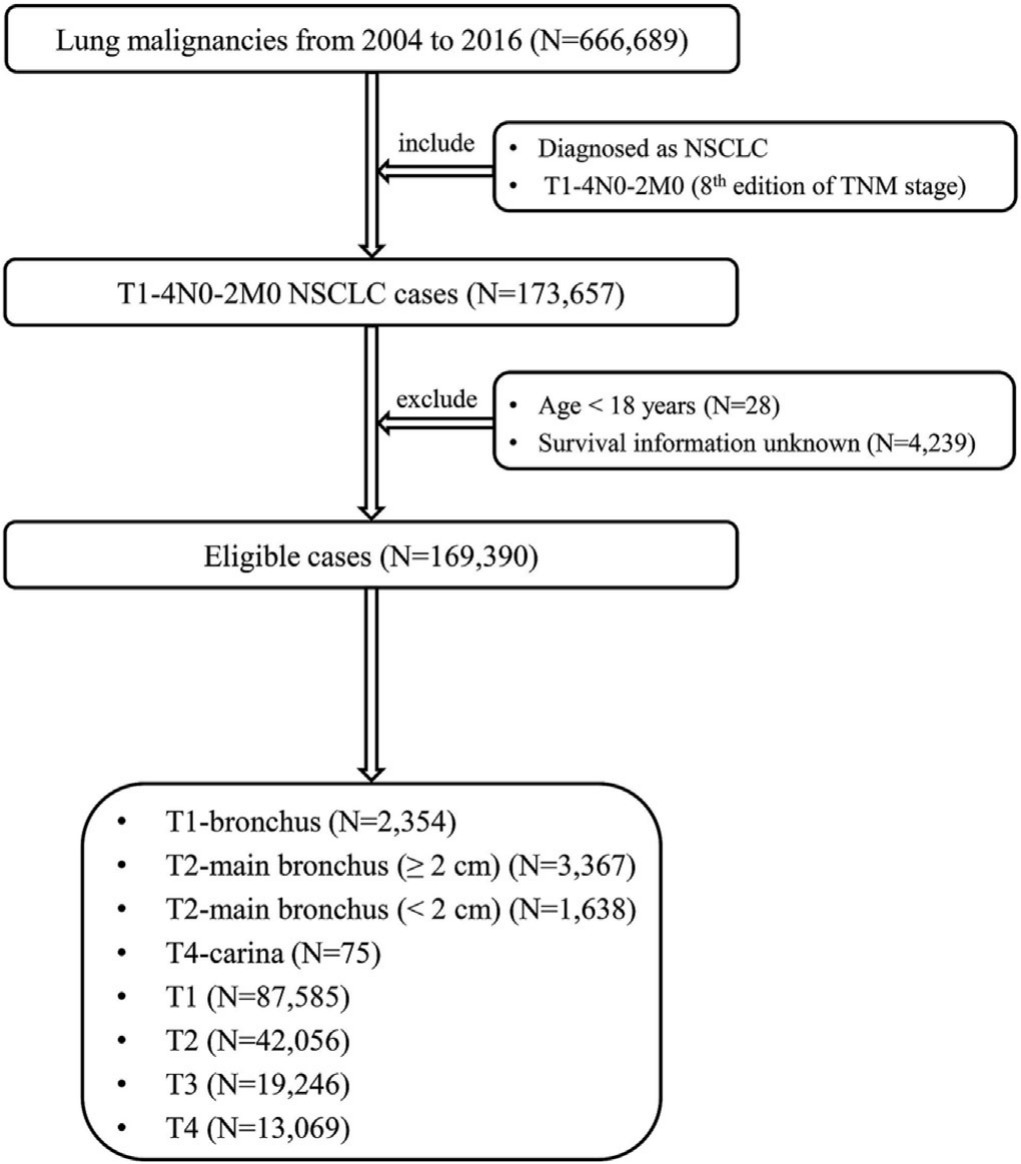
The patient selection flowchart of this study. T1‐bronchus, T1 superficial tumor of any size with invasive component limited to bronchial wall, with or without proximal extension to the main stem bronchus; T2‐main bronchus (≥2 cm), T2 tumors involving main stem bronchus ≥2.0 cm from carina; T2‐main bronchus (<2 cm), T2 tumors involving main stem bronchus <2.0 cm from carina; T4‐carina, T4 tumors with carina invasion; TNM, tumor‐node‐metastasis; NSCLC, non‐small cell lung cancer.

The data of lung malignancies between 2004 and 2016 were extracted from the SEER program (http://seer.cancer.gov). The inclusion criteria mandated that patients were diagnosed with NSCLC, and the TNM stages of tumors were T1‐4N0‐2M0. The exclusion criteria were age <18 years and unavailable survival information. The eligible patients were further separated into eight groups: T1‐bronchus (CS Extension code 110), T2‐main bronchus (≥2 cm) (CS Extension code 220), T2‐main bronchus (<2 cm) (CS Extension code 500), T4‐carina (CS Extension code 250), remaining T1, remaining T2, remaining T3 and remaining T4 group. The patient selection flowchart is showed in Figure 1. We further distinguished pathologically confirmed NSCLC from clinically confirmed ones based on the CS Tumor Size/Ext Eval codes (2.3 and 6) and the CS Lymph Nodes Eval codes (2.3 and 6).

### 2.2 Ethic

The author was authorized to retrieve the NSCLC data deposited in the SEER program with the reference number 12962‐Nov2019. This study was conducted in accordance with Helsinki declaration. This study was waived of personal‐informed consent forms because only anonymous data were used.

### 2.3 Data collection

The demographic and clinicopathological data, including age (continue, years), gender (male and female), race (Caucasian, African, and other), married status (other and married), insurance (without and with), tumor location (upper lobe, middle lobe, lower lobe, and other), surgery (not performed and performed), chemotherapy (not performed and performed), radiotherapy (not performed and performed), histology (adenocarcinoma, squamous cell carcinoma, and other), grade (I, II, III, and unknown), size (continue, mm), T category (T1‐bronchus, T2‐main bronchus [≥2 cm], T2‐main bronchus [<2 cm] and T4‐carina), and N category (N0, N1, and N2), were collected. The primary endpoint was overall survival (OS), which is defined as the time interval from the date of diagnosis to the date of death attributed to any cause or the last contact. The 8th edition of the NSCLC stage classification^5^ was used in this study.

### 2.4 Statistical analysis

R version 4.1.1 (The R Foundation for Statistical Computing, Vienna, Austria; http://www.r-project.org) and IBM SPSS Statistics version 25.0 (IBM Corp, Armonk, NY, USA) were used to conducted statistical analyses. The SEER*Stat software version 8.3.4. (http://seer.cancer.gov/seerstat) was used to extract the SEER datasheets. Non‐normally distributed continuous variables, presented as median (range), were compared using the Kruskal–Wallis H test and Mann–Whitney U test. Categorical variables, presented as numbers (percentages), were compared using the Pearson χ2 test. A forward stepwise univariable and multivariable Cox proportional hazard model was used to investigate the independent prognostic factors. The proportional hazards assumption was checked using the Schoenfeld residuals. C‐index was used to determine the best final Cox model. The Kaplan–Meier method was used to plot survival curves, the differences of which were compared using the log‐rank test. Propensity score matching (PSM)^9^ method was used to minimize the bias caused by the imbalanced baseline characteristics between groups. Two‐sided *P* values of <0.05 were considered statistically significant.

## 3 RESULTS

### 3.1 Baseline characteristic

A total of 169 390 eligible T1‐4N0‐2M0 NSCLC cases were included. There were 2354, 3367, 1638, 75, 87 585, 42 056, 19 246, and 13 069 cases in the T1‐bronchus, T2‐main bronchus (≥2 cm), T2‐main bronchus (<2 cm), T4‐carina, T1, T2, T3, and T4 groups, respectively. Regarding the patients with bronchus involvements (Table 1), there were more female (51.7%, *P* < 0.001), upper lobe tumors (57.2%, *P* < 0.001), and adenocarcinomas (57.5%, *P* < 0.001) in the T1‐bronchus group. In addition, patients in the T1‐bronchus group were inclined to receive surgery (76.7%, *P* < 0.001) rather than chemotherapy (20.4%, *P* < 0.001) and radiotherapy (6.9%, *P* < 0.001). More patients in the T2‐main bronchus (<2 cm) were younger (67 years, *P* = 0.003), received chemotherapy (62.7%, *P* < 0.001), and radiotherapy (16.6%, *P* < 0.001); were diagnosed as squamous cell carcinoma (70.9%, *P* < 0.001); and had regional lymph nodes metastasis (66.2%, *P* < 0.001). In the T4‐carina group, there were more male (69.3%, *P* < 0.001), Caucasians (89.3%, *P* = 0.001), and insured patients (68%, *P* < 0.001). After PSM, there were 122, 1362, 164, 601, and 61 pairs in the T2‐main bronchus (≥2 cm) and T2‐main bronchus (<2 cm), T1‐bronhucs and T1, T2‐main bronchus (≥2 cm) and T2, T2‐main bronchus (<2 cm), and T2 and T4‐carina and T4 matched cohorts, respectively. The baseline characteristics between groups were all balanced well after PSM (Tables S1–S5).

**TABLE 1:** The clinicopathological features of included patients.

Features	T1‐bronchus (*N* = 2354)	T2‐main bronchus (≥2 cm) (*N* = 3367)	T2‐main bronchus (<2 cm) (*N* = 1638)	T4‐carina (*N* = 75)	*P*
Age, years					0.003^a^
Continue	69 (18–94)	68 (24–94)	67 (19–97)	69 (44–85)	
Gender					<0.001
Male	1138 (48.3)	2022 (60.1)	1104 (67.4)	52 (69.3)	
Female	1216 (51.7)	1345 (39.9)	534 (32.6)	23 (30.7)	
Race					0.001
Caucasian	2063 (87.6)	2875 (85.4)	1357 (82.8)	67 (89.3)	
African	197 (8.4)	367 (10.9)	195 (11.9)	6 (8.0)	
Other	94 (4.0)	125 (3.7)	86 (5.3)	2 (2.7)	
Married status					0.349
Other	1056 (44.9)	1476 (43.8)	760 (46.4)	31 (41.3)	
Married	1298 (55.1)	1891 (56.2)	878 (53.6)	44 (58.7)	
Insurance					<0.001
Without	833 (35.4)	1396 (41.5)	783 (47.8)	24 (32.0)	
With	1521 (64.6)	1971 (58.5)	855 (52.2)	51 (68.0)	
Tumor location					<0.001
Upper lobe	1346 (57.2)	1790 (53.2)	831 (50.7)	33 (44.0)	
Middle lobe	144 (6.1)	139 (4.1)	40 (2.4)	1 (1.3)	
Lower lobe	760 (32.3)	1083 (32.2)	303 (18.5)	12 (16.0)	
Other	104 (4.4)	355 (10.5)	464 (28.3)	29 (38.7)	
Surgery					<0.001
Not performed	549 (23.3)	1272 (37.8)	1218 (74.4)	59 (78.7)	
Performed	1805 (76.7)	2095 (62.2)	420 (25.6)	16 (21.3)	
Chemotherapy					<0.001
Not performed	1874 (79.6)	2001 (59.4)	611 (37.3)	33 (44.0)	
Performed	480 (20.4)	1366 (40.6)	1027 (62.7)	42 (56.0)	
Radiotherapy					<0.001
Not performed	2191 (93.1)	2957 (87.8)	1366 (83.4)	69 (92.0)	
Performed	163 (6.9)	410 (12.2)	272 (16.6)	6 (8.0)	
Histology					<0.001
Adenocarcinoma	1354 (57.5)	1362 (40.5)	382 (23.3)	26 (34.7)	
Squamous cell carcinoma	771 (32.8)	1684 (50.0)	1161 (70.9)	47 (62.7)	
Other	229 (9.7)	321 (9.5)	95 (5.8)	2 (2.7)	
Grade					<0.001
I	376 (16.0)	250 (7.4)	68 (4.2)	7 (9.3)	
II	872 (37.0)	1138 (33.8)	425 (25.9)	15 (20.0)	
III	667 (28.3)	1254 (37.2)	578 (35.3)	28 (37.3)	
Unknown	439 (18.6)	725 (21.5)	567 (34.6)	25 (33.3)	
Size, mm					<0.001^a^
Continue	20 (1–240)	36 (3–960)	41 (3–850)	30 (10–790)	
*N* category					<0.001
0	1841 (78.2)	1834 (54.5)	553 (33.8)	36 (48.0)	
1	227 (9.6)	593 (17.6)	294 (17.9)	6 (8.0)	
2	286 (12.1)	940 (27.9)	791 (48.3)	33 (44.0)	

Abbreviations: T1‐bronchus, T1 superficial tumor of any size with invasive component limited to bronchial wall, with or without proximal extension to the main stem bronchus; T2‐main bronchus (≥2 cm), T2 tumors involving main stem bronchus ≥2.0 cm from carina; T2‐main bronchus (<2 cm), T2 tumors involving main stem bronchus <2.0 cm from carina; T4‐carina, T4 tumors with carina invasion.

^a^ Kruskal–Wallis H test.

### 3.2 Univariable and multivariable Cox analysis

Regarding the NSCLC patients with bronchus involvements, univariable Cox analysis indicated that age, gender, race, married status, insurance, tumor location, surgery, chemotherapy, radiotherapy, histology, grade, visceral pleural invasion, tumor size, T category, and N category were potential prognostic factors (Table 2). In further analyses, multivariable Cox analysis confirmed that age (*P* < 0.001), gender (*P* < 0.001), married status (*P* < 0.001), insurance (*P* < 0.001), tumor location (*P* = 0.008), surgery (*P* < 0.001), chemotherapy (*P* < 0.001), histology (*P* = 0.001), grade (*P* < 0.001), tumor size (*P* < 0.001), T category (HR: T1‐bronchus vs. T2‐main bronchus [≥2 cm] vs. T2‐main bronchus [<2 cm] vs. T4‐carina = 1 vs. 1.204 vs. 1.298 vs. 1.315, *P* < 0.001) and N category (*P* < 0.001) were independent prognostic factors (Table 2).

**TABLE 2:** The Cox analyses of overall survival in the patients with bronchus involvements.

Prognostic variables	Univariable			Multivariable		
	HR	95% CI	*P*	HR	95% CI	*P*
Age, years			<0.001			<0.001
Continue	1.030	1.027–1.033		1.023	1.020–1.026	
Gender			<0.001			<0.001
Male	1			1		
Female	0.727	0.687–0.769		0.800	0.754–0.850	
Race			0.019			0.560
Caucasian	1			1		
African	1.121	1.026–1.224		0.995	0.909–1.089	
Other	0.926	0.800–1.071		0.923	0.798–1.068	
Married status			<0.001			<0.001
Other	1			1		
Married	0.838	0.793–0.885		0.873	0.824–0.924	
Insurance			<0.001			<0.001
Without	1			1		
With	0.863	0.816–0.912		0.838	0.792–0.886	
Tumor location			<0.001			0.008
Upper lobe	1			1		
Middle lobe	0.957	0.831–1.101		1.179	1.024–1.357	
Lower lobe	1.024	0.961–1.090		1.079	1.012–1.150	
Other	1.457	1.342–1.581		1.104	1.014–1.203	
Surgery			<0.001			<0.001
Not performed	1			1		
Performed	0.301	0.285–0.319		0.359	0.334–0.387	
Chemotherapy			<0.001			<0.001
Not performed	1			1		
Performed	1.171	1.108–1.237		0.672	0.629–0.718	
Radiotherapy			0.146			
Not performed	1					
Performed	1.064	0.978–1.157				
Histology			<0.001			0.001
Adenocarcinoma	1			1		
Squamous cell carcinoma	1.566	1.478–1.658		1.118	1.050–1.190	
Other	0.909	0.811–1.018		0.990	0.882–1.110	
Grade			<0.001			<0.001
I	1			1		
II	1.509	1.343–1.696		1.226	1.088–1.382	
III	1.843	1.642–2.069		1.356	1.203–1.528	
Unknown	2.431	2.158–2.737		1.142	1.008–1.295	
Visceral pleural invasion			0.393			‐
Without	1			‐		
With	0.706	0.317–1.571		‐	‐	‐
Size			<0.001			<0.001
Continue	1.004	1.003–1.004		1.002	1.001–1.002	
T category			<0.001			<0.001
T1‐bronchus	1			1		
T2‐main bronchus (≥2 cm)	1.522	1.423–1.627		1.204	1.121–1.293	
T2‐main bronchus (<2 cm)	2.336	2.164–2.521		1.298	1.185–1.422	
T4‐carina	2.300	1.775–2.979		1.315	1.010–1.712	
*N* category			<0.001			<0.001
0	1			1		
1	1.316	1.216–1.424		1.384	1.273–1.505	
2	2.167	2.038–2.304		1.567	1.457–1.685	

Abbreviations: T1‐bronchus, T1 superficial tumor of any size with invasive component limited to bronchial wall, with or without proximal extension to the main stem bronchus; T2‐main bronchus (≥2 cm), T2 tumors involving main stem bronchus ≥2.0 cm from carina; T2‐main bronchus (<2 cm), T2 tumors involving main stem bronchus <2.0 cm from carina; T4‐carina, T4 tumors with carina invasion.

Multivariable Cox analysis of T category in the subgroups of the NSCLC patients with bronchus involvements (Table 3) showed that T category was an independent prognostic factor in the pathological TNM stage group (HR: T1‐bronchus vs. T2‐main bronchus [≥2 cm] vs. T2‐main bronchus [<2 cm] vs. T4‐carina = 1 vs. 1.210 vs. 1.302 vs. 0.961, *P* < 0.001), in the clinical TNM stage group (HR: T1‐bronchus vs. T2‐main bronchus [≥2 cm] vs. T2‐main bronchus [<2 cm] vs. T4‐carina = 1 vs. 1.153 vs. 1.306 vs. 1.244, *P* < 0.001), in the N0 subgroup (HR: T1‐bronchus vs. T2‐main bronchus [≥2 cm] vs. T2‐main bronchus [<2 cm] vs. T4‐carina = 1 vs. 1.193 vs. 1.282 vs. 1.285, *P* < 0.001) and in the resected subgroup (HR: T1‐bronchus vs. T2‐main bronchus [≥2 cm] vs. T2‐main bronchus [<2 cm] vs. T4‐carina = 1 vs. 1.223 vs. 1.190 vs. 0.996, *P* < 0.001).

**TABLE 3:** Multivariable Cox analyses of T category in the subgroups of the patients with bronchus involvements.

Pathological subgroup	HR	95% CI	*P*
T category			0.001
T1‐bronchus	1		
T2‐main bronchus (≥2 cm)	1.210	1.093–1.340	
T2‐main bronchus (<2 cm)	1.302	1.090–1.555	
T4‐carina	0.961	0.455–2.029	

Clinical subgroup	HR	95% CI	*P*
T category			<0.001
T1‐bronchus	1		
T2‐main bronchus (≥2 cm)	1.153	1.030–1.290	
T2‐main bronchus (<2 cm)	1.306	1.156–1.476	
T4‐carina	1.244	0.926–1.672	

N0 subgroup	HR	95% CI	*P*
T category			<0.001
T1‐bronchus	1		
T2‐main bronchus (≥2 cm)	1.193	1.091–1.305	
T2‐main bronchus (<2 cm)	1.282	1.117–1.472	
T4‐carina	1.285	0.871–1.896	

Resected subgroup	HR	95% CI	*P*
T category			<0.001
T1‐bronchus	1		
T2‐main bronchus (≥2 cm)	1.223	1.116–1.340	
T2‐main bronchus (<2 cm)	1.190	1.023–1.384	
T4‐carina	0.996	0.533–1.863	

Abbreviations: T1‐bronchus, T1 superficial tumor of any size with invasive component limited to bronchial wall, with or without proximal extension to the main stem bronchus; T2‐main bronchus (≥2 cm), T2 tumors involving main stem bronchus ≥2.0 cm from carina; T2‐main bronchus (<2 cm), T2 tumors involving main stem bronchus <2.0 cm from carina; T4‐carina, T4 tumors with carina invasion.

### 3.3 Survival analysis

**FIGURE 2:**
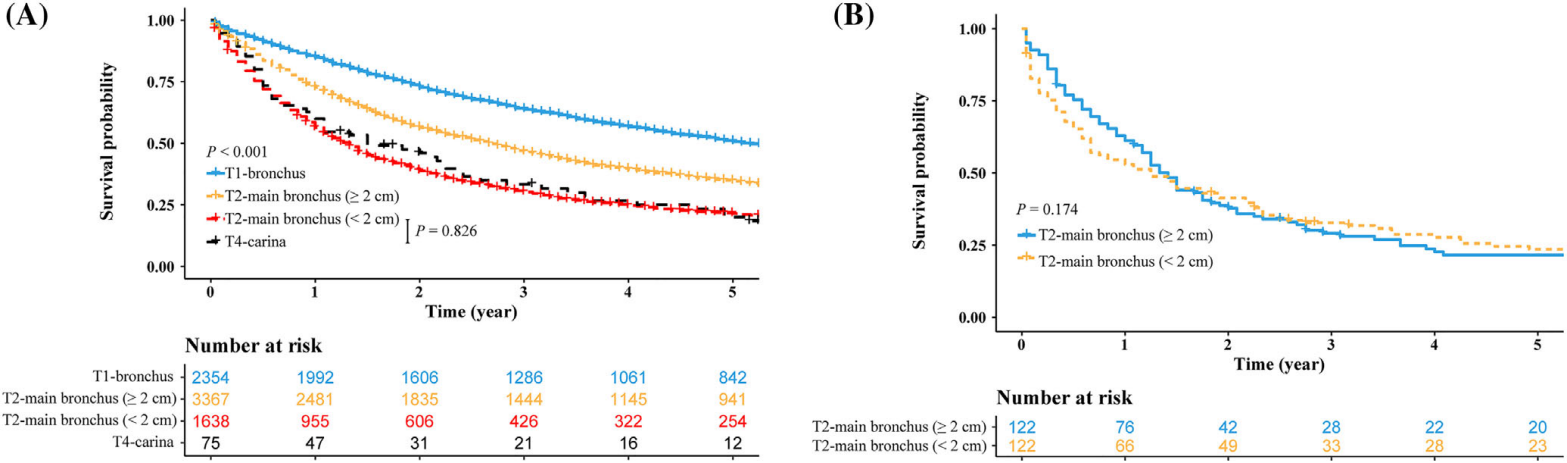
The survival comparisons among patients with bronchus involvements. (A) T1‐bronchus versus T2‐main bronchus (≥2 cm) versus T2‐main bronchus (<2 cm) versus T4‐carina and (B) T2‐main bronchus (≥2 cm) versus T2‐main bronchus (<2 cm) after PSM. T1‐bronchus, T1 superficial tumor of any size with invasive component limited to bronchial wall, with or without proximal extension to the main stem bronchus; T2‐main bronchus (≥2 cm), T2 tumors involving main stem bronchus ≥2.0 cm from carina; T2‐main bronchus (<2 cm), T2 tumors involving main stem bronchus <2.0 cm from carina; T4‐carina, T4 tumors with carina invasion; PSM, propensity score matching.

The 3‐year OS rates of the T1‐bronchus, T2‐main bronchus (≥2 cm), T2‐main bronchus (<2 cm), and T4‐carina patients were 63.7%, 46.5%, 30.2%, and 33.3%, respectively (Figure 2A). The 5‐year OS rates were 50.7%, 34.6%, 21.5%, and 20.0%, respectively (Figure 2A). T1‐bronchus patients had the best survival rates, followed by T2‐main bronchus (≥2 cm) patients. T2‐main bronchus (<2 cm) patients and T4‐carina patients had similar survival rates (*P* = 0.826; Figure 2A). In the T2‐main bronchus (≥2 cm) and T2‐main bronchus (<2 cm) matched cohort, the survivals of T2‐main bronchus (≥2 cm) patients were comparable to those of T2‐main bronchus (<2 cm) patients (*P* = 0.174; Figure 2B).

**FIGURE 3:**
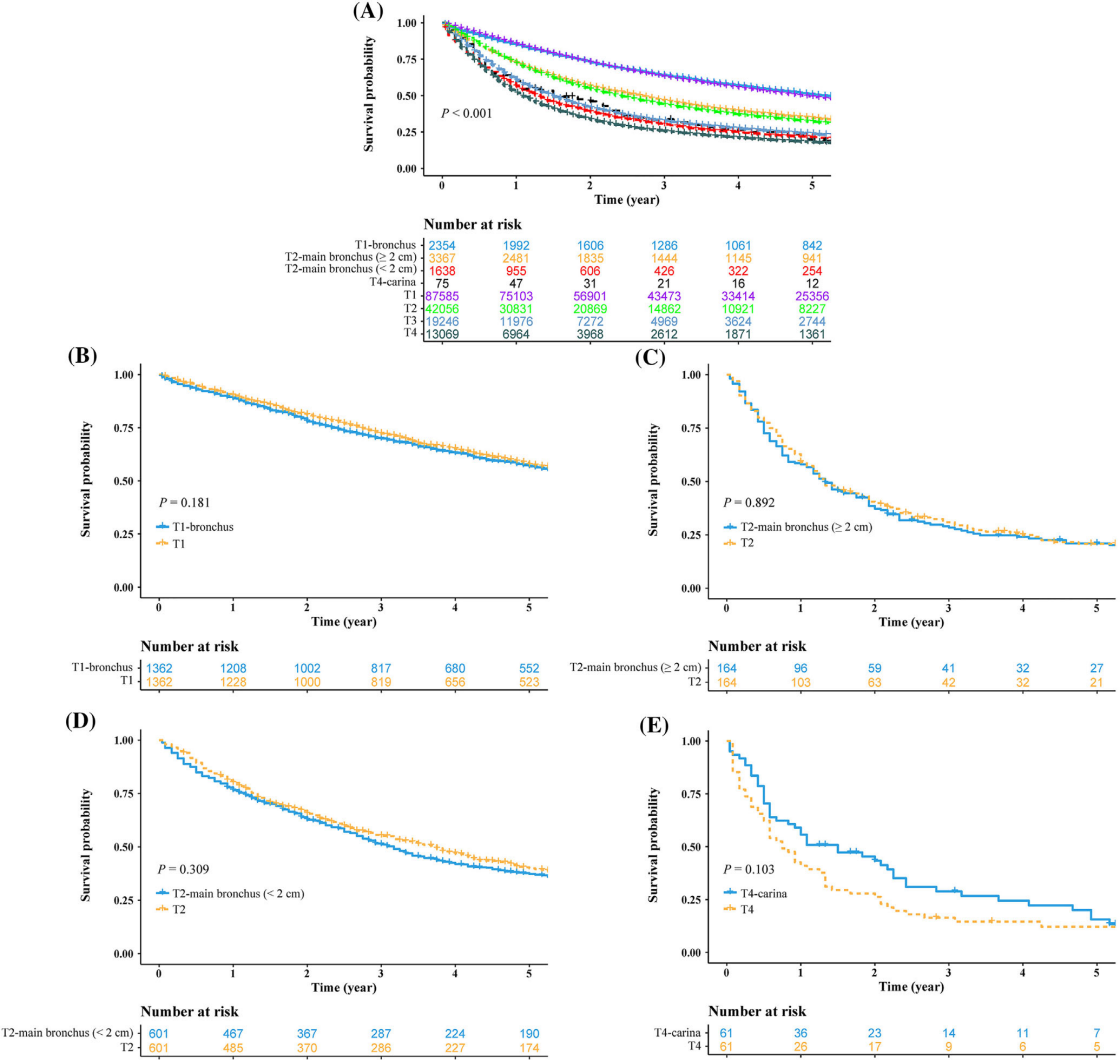
The survival comparisons between the patients with bronchus involvements and the remaining T1‐T4 patients. (A) T1‐bronchus versus T2‐main bronchus (≥2 cm) versus T2‐main bronchus (<2 cm) versus T4‐carina versus T1 versus T2 versus T3 versus T4; (B) T1‐bronchus versus T1 after PSM; (C) T2‐main bronchus (≥2 cm) versus T2 after PSM; (D) T2‐main bronchus (<2 cm) versus T2 after PSM; and (E) T4‐carina versus T4 after PSM. T1‐bronchus, T1 superficial tumor of any size with invasive component limited to bronchial wall, with or without proximal extension to the main stem bronchus; T2‐main bronchus (≥2 cm), T2 tumors involving main stem bronchus ≥2.0 cm from carina; T2‐main bronchus (<2 cm), T2 tumors involving main stem bronchus <2.0 cm from carina; T4‐carina, T4 tumors with carina invasion; PSM, propensity score matching.

When compared with the remaining T1–T4 patients, T1‐bronchus patients and T1 patients had almost overlapping survival curves (*P* = 0.142; Figure 3A). The survivals of T2‐main bronchus (≥2 cm) patients were superior to those of T2 patients (*P* = 0.001; Figure 3A). T2‐main bronchus (<2 cm) patients had worse survivals than both T2 patients (*P* < 0.001; Figure 3A) and T3 patients (*P* < 0.001; Figure 3A). Furthermore, the survivals of T4‐carina patient were comparable to those of T2‐main bronchus (<2 cm) patients (*P* = 0.597; Figure 3A) and T4 patients (*P* = 0.279; Figure 3A).

After PSM, in the T1‐bronchus and T1 matched cohort, bronchus invasion did not confer an inferior survival when compared with the remaining T1 patients (*P* = 0.181; Figure 3B). In the T2‐main bronchus (≥2 cm) and T2 matched cohort, the survival curve of T2‐main bronchus (≥2 cm) patients nearly overlapped with that of T2 patients (*P* = 0.892; Figure 3C). In the T2‐main bronchus (<2 cm) and T2 matched cohort, it is interesting to observe that these two groups of patients had similar survivals (*P* = 0.309; Figure 3D). In the T4‐carina and T4 matched cohort, the survivals of T4‐carina patients were better than those of T4 patients, but the difference was not statistically significant (*P* = 0.103; Figure 3E).

## 4 DISCUSSION

Previously, many researches has been focused on the validations and refinements of the current 8th edition of the NSCLC TNM stage classification.^10^, ^11^, ^12^, ^13^, ^14^ To date, there was no validation study focused on the T category of the NSCLC patients with bronchus involvements. Herein, we analyzed the data of NSCLC patients with bronchus involvements and aimed to externally validate the current TNM stage classification. Our study demonstrated that T1‐bronchus patients had the best prognosis; T2‐main bronchus (≥2 cm) and T2‐main bronchus (<2 cm) patients had similar prognosis both in the entire cohort and in several subgroups. Survival curves further showed that T1‐bronchus and T1 patients had comparable survival rates; the survivals of T2‐main bronchus patients regardless of the distance from carina were similar to those of T2 patients, and the survivals of T4‐carina patients were also similar to those of T4 patients. Our results validated and supported the current TNM stage classification, and we proposed that it might not be necessary to modify T categories of the NSCLC patients with bronchus involvements in the next version of the NSCLC TNM stage classification.

In the proposals for the revisions of the T descriptors in the 8th edition of the lung cancer TNM classification,^6^ Rami‐Porta et al.^7^ reported that tumors involving main bronchus ≥2 cm from carina are still defined as T2 category, and those involving main bronchus <2 cm from carina, a T3 descriptor in the 7th edition of the TNM classification,^7^, ^8^ should be downstaged from T3 to T2 category. Regarding patients with tumors of any size with invasive component limited to bronchial wall and patients with tumors with carina involvement, detailed descriptions were not provided. There is a dearth of study focused on the patients with bronchus involvements partially because of the small number of cases. Therefore, external validations are needed.

With the advantage provided by the large data deposited in the SEER program, the current study was sufficient to investigate the prognostic differences among these patients. Our results showed that the survivals of T1‐bronchus patients were comparable to those of T1 patients; T2‐main bronchus patients regardless of the distance from carina and T2 patients also had similar survival rates; and the survivals of T4‐carina patients were similar to those of T4 patients. To the best of our knowledge, the current study is the first comprehensive analysis of the prognosis of patients with bronchus involvements. Our results supported the current T category of these patients and might provide certain reference value for the proposals of the T category in the next version of the NSCLC TNM stage classification.

Our study had several limitations. First, a relatively small number of cases in the T4‐carina group (75 cases) made it hard to draw a conclusion with strong statistical power. Therefore, large sample studies are encouraged to confirm the conclusions of this study. Second, in the era of target therapies and immunotherapies, whether our conclusions were applied to the patients received tyrosine kinase inhibitors or immune checkpoint inhibitors is unknown. It is interesting to further explore the prognosis disparities among these patients who had received novel therapies. However, the information about treatments regimens, dosage, and timing is unavailable in the SEER program. We planned to collected the related information in our institution and further validate our results in future. In addition, our results were exploratory because a large number of hypothesis tests were performed and multiplicity might exist. At last, this is a retrospective study, and bias cannot be avoided.

In conclusion, our results validated and supported the current T category of the patients with bronchus involvements, which might provide certain reference value for the revisions of T category in the next version of the NSCLC TNM stage classification.

## AUTHOR CONTRIBUTIONS

*Conception and design*: Gang Wang. *Administrative support*: Yong‐Qiang Ye. *Provision of study materials or patients*: Bao‐Long Xie, Xiang‐Min Lai, and Sheng‐Peng Zhong. *Collection and assembly of data*: Gang Wang. *Data analysis and interpretation*: Gang Wang. *Manuscript writing*: All authors. *Final approval of manuscript*: All authors.

## CONFLICT OF INTEREST

The author declare no conflict of interest.

## ETHICS STATEMENT

The Ethics Committee of Ganzhou Cancer Hospital approved this study. This study was conducted in accordance with the Declaration of Helsinki. This study utilized the de‐identified data deposited in the SEER database, and hence, the requirement for individual informed consent forms was waived.

## Supporting information


**Table S1.** The covariates between T2‐main bronchus (≥ 2 cm) and T2‐main bronchus (< 2 cm) patients after PSM
**Table S2.** The covariates between T1‐bronchus and T1 patients after PSM
**Table S3.** The covariates between T2‐main bronchus (≥ 2 cm) and T2 patients after PSM
**Table S4.** The covariates between T2‐main bronchus (< 2 cm) and T2 patients after PSM
**Table S5.** The covariates between T4‐carina and T4 patients after PSM

## Data Availability

The data are available in the Surveillance, Epidemiology, and End Results (SEER) database (at https://seer.cancer.gov/).
