# Supplementary material for: Investigation of the non‐small cell lung cancer patients with bronchus involvements: A population‐based study
Source: Clin Respir J. 2023 Aug 7;17(9):941–50. doi: 10.1111/crj.13683 (PMC10500318; doi:10.1111/crj.13683)
Supplement: Supplementary file 1 — Table S1. The covariates between T2‐main bronchus (≥ 2 cm) and T2‐main bronchus (< 2 cm) patients after PSM Table S2. The covariates between T1‐bronchus and T1 patients after PSM Table S3. The covariates between T2‐main bronchus (≥ 2 cm) and T2 patients after PSM Table S4. The covariates between T2‐main bronchus (< 2 cm) and T2 patients after PSM Table S5. The covariates between T4‐carina and T4 patients after PSM [file CRJ-17-941-s001.docx]

**Table S1. The covariates between T2-main bronchus (≥ 2 cm) and T2-main bronchus (< 2 cm) patients after PSM**

| Covariates | T2-main bronchus (≥ 2 cm)  (N= 122) | T2-main bronchus (< 2 cm)  (N= 122) | *P* |
| --- | --- | --- | --- |
| Age, years |  |  | 0.689^a^ |
| Continue | 70 (44-89) | 70 (42-89) |  |
| Gender |  |  | 0.777 |
| Male | 86 (70.5) | 88 (72.1) |  |
| Female | 36 (29.5) | 34 (27.9) |  |
| Surgery |  |  | 0.893 |
| Not performed | 79 (64.8) | 80 (65.6) |  |
| Performed | 43 (35.2) | 42 (34.4) |  |
| Chemotherapy |  |  | 0.199 |
| Not performed | 60 (49.2) | 70 (57.4) |  |
| Performed | 62 (50.8) | 52 (42.6) |  |
| Radiotherapy |  |  | 0.436 |
| Not performed | 105 (86.1) | 109 (89.3) |  |
| Performed | 17 (13.9) | 13 (10.7) |  |
| Histology |  |  | 0.521 |
| Adenocarcinoma | 33 (27.0) | 26 (21.3) |  |
| Squamous cell carcinoma | 84 (68.9) | 92 (75.4) |  |
| Other | 5 (4.1) | 4 (3.3) |  |
| Grade |  |  | 0.084 |
| I | 5 (4.1) | 0 (0.0) |  |
| II | 40 (32.8) | 44 (36.1) |  |
| III | 35 (28.7) | 43 (35.2) |  |
| Unknown | 42 (34.4) | 35 (28.7) |  |
| Size, mm |  |  | 0.912^a^ |
| Continue | 30.0 | 30.0 |  |
| N category |  |  | 0.513 |
| 0 | 52 (42.6) | 45 (36.9) |  |
| 1 | 19 (15.6) | 25 (20.5) |  |
| 2 | 51 (41.8) | 52 (42.6) |  |

a Mann-Whitney U test

T2-main bronchus (≥ 2 cm), T2 tumors involving main stem bronchus greater than or equal to 2.0 cm from carina; T2-main bronchus (< 2 cm), T2 tumors involving main stem bronchus less than 2.0 cm from carina; PSM, propensity score matching

**Table S2. The covariates between T1-bronchus and T1 patients after PSM**

| Covariates | T1-bronchus  (N=1,362) | T1  (N=1,362) | *P* |
| --- | --- | --- | --- |
| Age, years |  |  | 0.838^a^ |
| Continue | 69 (23-94) | 69 (23-94) |  |
| Gender |  |  | 0.969 |
| Male | 639 (46.9) | 640 (47.0) |  |
| Female | 723 (53.1) | 722 (53.0) |  |
| Surgery |  |  | 0.797 |
| Not performed | 230 (16.9) | 225 (16.5) |  |
| Performed | 1,132 (83.1) | 1,137 (83.5) |  |
| Chemotherapy |  |  | 1.000 |
| Not performed | 1,267 (93.0) | 1,267 (93.0) |  |
| Performed | 95 (7.0) | 95 (7.0) |  |
| Radiotherapy |  |  | 1.000 |
| Not performed | 1,344 (98.7) | 1,344 (98.7) |  |
| Performed | 18 (1.3) | 18 (1.3) |  |
| Histology |  |  | 0.885 |
| Adenocarcinoma | 895 (65.7) | 893 (65.6) |  |
| Squamous cell carcinoma | 389 (28.6) | 385 (28.3) |  |
| Other | 78 (5.7) | 84 (6.2) |  |
| Grade |  |  | 0.999 |
| I | 236 (17.3) | 239 (17.5) |  |
| II | 553 (40.6) | 552 (40.5) |  |
| III | 354 (26.0) | 353 (25.9) |  |
| Unknown | 219 (16.1) | 218 (16.0) |  |
| Size, mm |  |  | 0.858^a^ |
| Continue | 19.0 | 18.5 |  |
| N category |  |  | 1.000 |
| 0 | 1,276 (93.7) | 1,276 (93.7) |  |
| 1 | 34 (2.5) | 34 (2.5) |  |
| 2 | 52 (3.8) | 52 (3.8) |  |

a Mann-Whitney U test

T1-bronchus, T1 superficial tumor of any size with invasive component limited to bronchial wall, with or without proximal extension to the main stem bronchus; PSM, propensity score matching

**Table S3. The covariates between T2-main bronchus (≥ 2 cm) and T2 patients after PSM**

| Covariates | T2-main bronchus (≥ 2 cm)  (N=164) | T2  (N=164) | *P* |
| --- | --- | --- | --- |
| Age, years |  |  | 0.901^a^ |
| Continue | 70 (48-88) | 70 (48-88) |  |
| Gender |  |  | 0.635 |
| Male | 114 (69.5) | 110 (67.1) |  |
| Female | 50 (30.5) | 54 (32.9) |  |
| Surgery |  |  | 0.900 |
| Not performed | 121 (73.8) | 120 (73.2) |  |
| Performed | 43 (26.2) | 44 (26.8) |  |
| Chemotherapy |  |  | 0.821 |
| Not performed | 63 (38.4) | 65 (39.6) |  |
| Performed | 101 (61.6) | 99 (60.4) |  |
| Radiotherapy |  |  | 0.683 |
| Not performed | 150 (91.5) | 152 (92.7) |  |
| Performed | 14 (8.5) | 12 (7.3) |  |
| Histology |  |  | 0.881 |
| Adenocarcinoma | 34 (20.7) | 37 (22.6) |  |
| Squamous cell carcinoma | 125 (76.2) | 123 (75.0) |  |
| Other | 5 (3.0) | 4 (2.4) |  |
| Grade |  |  | 0.997 |
| I | 2 (1.2) | 2 (1.2) |  |
| II | 42 (25.6) | 41 (25.0) |  |
| III | 57 (34.8) | 56 (34.1) |  |
| Unknown | 63 (38.4) | 65 (39.6) |  |
| Size, mm |  |  | 0.686^a^ |
| Continue | 42.0 | 42.0 |  |
| N category |  |  | 0.982 |
| 0 | 64 (39.0) | 65 (39.6) |  |
| 1 | 18 (11.0) | 17 (10.4) |  |
| 2 | 82 (50.0) | 82 (50.0) |  |

a Mann-Whitney U test

T2-main bronchus (≥ 2 cm), T2 tumors involving main stem bronchus greater than or equal to 2.0 cm from carina; PSM, propensity score matching

**Table S4. The covariates between T2-main bronchus (< 2 cm) and T2 patients after PSM**

| Covariates | T2-main bronchus (< 2 cm)  (N=601) | T2  (N=601) | *P* |
| --- | --- | --- | --- |
| Age, years |  |  | 0.803^a^ |
| Continue | 71 (35-90) | 71 (35-90) |  |
| Gender |  |  | 1.000 |
| Male | 376 (62.6) | 376 (62.6) |  |
| Female | 225 (37.4) | 225 (37.4) |  |
| Surgery |  |  | 0.950 |
| Not performed | 181 (30.1) | 180 (30.0) |  |
| Performed | 420 (69.9) | 421 (70.0) |  |
| Chemotherapy |  |  | 0.649 |
| Not performed | 436 (72.5) | 443 (73.7) |  |
| Performed | 165 (27.5) | 158 (26.3) |  |
| Radiotherapy |  |  | 0.777 |
| Not performed | 574 (95.5) | 576 (95.8) |  |
| Performed | 27 (4.5) | 25 (4.2) |  |
| Histology |  |  | 0.998 |
| Adenocarcinoma | 270 (44.9) | 271 (45.1) |  |
| Squamous cell carcinoma | 314 (52.2) | 313 (52.1) |  |
| Other | 17 (2.8) | 17 (2.8) |  |
| Grade |  |  | 0.986 |
| I | 40 (6.7) | 39 (6.5) |  |
| II | 233 (38.8) | 231 (38.4) |  |
| III | 221 (36.8) | 219 (36.4) |  |
| Unknown | 107 (17.8) | 112 (18.6) |  |
| Size, mm |  |  | 0.844^a^ |
| Continue | 40.0 | 40.0 |  |
| N category |  |  | 0.993 |
| 0 | 438 (72.9) | 438 (72.9) |  |
| 1 | 57 (9.5) | 58 (9.7) |  |
| 2 | 106 (17.6) | 105 (17.5) |  |

a Mann-Whitney U test

T2-main bronchus (< 2 cm), T2 tumors involving main stem bronchus less than 2.0 cm from carina; PSM, propensity score matching

**Table S5. The covariates between T4-carina and T4 patients after PSM**

| Covariates | T4-carina  (N=61) | T4  (N=61) | *P* |
| --- | --- | --- | --- |
| Age, years |  |  | 0.760^a^ |
| Continue | 70 (51-85) | 71 (51-85) |  |
| Gender |  |  | 0.686 |
| Male | 43 (70.5) | 45 (73.8) |  |
| Female | 18 (29.5) | 16 (26.2) |  |
| Surgery |  |  | 1.000 |
| Not performed | 53 (86.9) | 53 (86.9) |  |
| Performed | 8 (13.1) | 8 (13.1) |  |
| Chemotherapy |  |  | 1.000 |
| Not performed | 26 (42.6) | 26 (42.6) |  |
| Performed | 35 (57.4) | 35 (57.4) |  |
| Radiotherapy |  |  | 0.464 |
| Not performed | 58 (95.1) | 56 (91.8) |  |
| Performed | 3 (4.9) | 5 (8.2) |  |
| Histology |  |  | 0.926 |
| Adenocarcinoma | 20 (32.8) | 18 (29.5) |  |
| Squamous cell carcinoma | 40 (65.6) | 42 (68.9) |  |
| Other | 1 (1.6) | 1 (1.6) |  |
| Grade |  |  | 0.996 |
| I | 5 (8.2) | 5 (8.2) |  |
| II | 11 (18.0) | 12 (19.7) |  |
| III | 22 (36.1) | 22 (36.1) |  |
| Unknown | 23 (37.7) | 22 (36.1) |  |
| N category |  |  | 0.934 |
| 0 | 28 (45.9) | 30 (49.2) |  |
| 1 | 2 (3.3) | 2 (3.3) |  |
| 2 | 31 (50.8) | 29 (47.5) |  |

a Mann-Whitney U test

T4-carina, T4 tumors with carina invasion; PSM, propensity score matching
